# Exploring the TyG Index and the Homeostasis Model Assessment of Insulin Resistance as Insulin Resistance Markers: Implications for Fibromyalgia Management and Understanding—A Narrative Review

**DOI:** 10.3390/diagnostics15040494

**Published:** 2025-02-18

**Authors:** Amirsaeed Samavarchitehrani, Filiz Mercantepe, Amir Hossein Behnoush, Aleksandra Klisic

**Affiliations:** 1 Faculty of Medicine, Islamic Azad University, Tehran 1474833163, Iran; 2 Department of Endocrinology and Metabolism, Faculty of Medicine, Recep Tayyip Erdogan University, Rize 53100, Turkey; filiz.mercantepe@saglik.gov.tr; 3 School of Medicine, Tehran University of Medical Sciences, Tehran 1474833163, Iran; amirhossein.behnoush@gmail.com; 4 Faculty of Medicine, University of Montenegro, 81000 Podgorica, Montenegro; aleksandranklisic@gmail.com; 5 Center for Laboratory Diagnostics, Primary Health Care Center, 81000 Podgorica, Montenegro

**Keywords:** fibromyalgia, insulin resistance, triglyceride–glucose index, HOMA-IR

## Abstract

Fibromyalgia (FM) is a chronic musculoskeletal disease with a higher prevalence among women. To date, there has been no definitive laboratory or imaging assessment for FM, and hence, the diagnosis criteria for FM remained based on subjective assessment of symptoms with high overlap with other rheumatological disorders. Many patients with FM suffer from metabolic disorders leading to insulin resistance (IR). There have been several methods to assess IR, among which the Homeostasis Model Assessment of Insulin Resistance (HOMA-IR) and the triglyceride–glucose (TyG) index have been used more frequently, with the latter being more available and cost-effective. As higher IR has been reported for patients with FM with various mechanisms, in this review, we sought to investigate the association between IR and FM using the current evidence. One of the possible underlying mechanisms of this association might be mitochondrial dysfunction and oxidative stress observed in IR conditions and its role in FM. Studies have also shown that IR indices are higher in patients with FM, compared to healthy controls, while higher HOMA-IR levels were also reported for higher severities of FM based on Fibromyalgia Impact Questionnaire—Revised (FIQR) scores. While these findings suggest the possible involvement of IR in FM pathophysiology and add to the value of IR measurement in FM clinical assessment, further large-scale studies are needed to establish a definitive causal association between them.

## 1. Introduction

Fibromyalgia (FM) is a chronic disease defined by widespread pain resulting from improper processing of pain signals [1]. The prevalence of FM is estimated at about 2% to 4% of the worldwide population, while it is more common in women [2]. Many clinical complaints have been explored for FM, such as multiple tender points, persistent fatigue, sleep disturbances, mood changes, memory issues, and difficulties with cognitive function [3,4]. While some biomarkers have been suggested for FM [5,6,7], currently, there are no dependable laboratory assessments that can accurately diagnose FM [8]. As a result, the criteria used to diagnose FM are still based on subjective assessments, with symptoms that are common and frequently overlap with those of other rheumatic conditions [9].

While factors such as genetic predisposition, negative life events, and physical trauma are acknowledged as contributing to the risk of developing FM, the exact cause remains unidentified [10,11]. However, chronic fatigue syndrome and other related diseases known as “central sensitivity syndromes” frequently coexist with FM. Pain boosters are believed to be caused by disrupted neuroplasticity. This might result in pain hypersensitivity due to the central nervous system’s processes being activated regardless of neural lesions or peripheral inflammation. Therefore, the possible involvement of neuroplasticity mediators in the underlying mechanisms of FM has been suggested [12,13,14,15]. Finally, microvascular dysfunction plays an important role in chronic pain that has been shown in several studies and through assessment of biomarkers involved in endothelial dysfunction [16,17,18].

Insulin resistance [IR] refers to a condition in which cells exhibit reduced sensitivity to insulin, resulting in elevated blood glucose levels. In response, the body produces more insulin, a phenomenon known as hyperinsulinemia [19]. The hyperinsulinemic–euglycemic clamp represents the most accurate approach for the evaluation of sensitivity to insulin. Nonetheless, it demands considerable time and effort, and it can be expensive and technically difficult to execute [20]. In addition, more available methods have been suggested for evaluating IR; the Homeostasis Model Assessment of Insulin Resistance (HOMA-IR) is a non-invasive method commonly used in research to measure insulin resistance [21]. Another tool for assessing IR is the triglyceride–glucose (TyG) index, which is a low-cost and easily available index, especially in settings where measurement of insulin is not accessible, such as in low-income countries [22]. Elevated glucose levels and IR have been shown to increase pain sensitivity and reduce the body’s ability to suppress pain. This relationship has been observed in both healthy individuals and patients with FM, suggesting that metabolic factors may contribute to altered pain processing in these populations [23,24]. Moreover, research has shown that people with FM generally exhibit elevated fasting insulin levels and increased IR when compared to those who are healthy [25]. Hence, understanding the relationship between IR and FM would provide valuable therapeutic opportunities.

Despite the increasing recognition of the potential link between FM and IR, this association remains inadequately explored. Existing studies are limited, and the mechanisms connecting metabolic dysfunction to FM symptoms are not fully understood. Exploring these links may determine better management strategies. This narrative review aims to fill this gap and summarize current evidence.

## 2. Methods

### 2.1. Search Strategy and Study Selection

We performed a comprehensive search across three primary databases: PubMed, Scopus, and Web of Science. This involved the use of a combination of Medical Subject Headings (MeSH) terms alongside other pertinent keywords to find all relevant studies published until 10 December 2024. The specific search strategy is outlined in Supplementary Table S1.

We included studies that (1) assessed the association between the TyG index OR HOMA-IR and FM, (2) evaluated the TyG index OR HOMA-IR in individuals with FM (alongside a control group), and (3) compared TyG levels OR HOMA-IR based on the severity of FM. The studies that were excluded were (1) conference abstracts, (2) case reports, and (3) articles not published in English. Conference abstracts and case reports were excluded due to their small sample sizes, lack of control groups, and lack of peer-reviewed validation. The diagnosis of FM was determined based on the criteria established by the American College of Rheumatology in 1990 [4].

The records were combined, and duplicate entries were eliminated using EndNote. Next, two separate reviewers (AS and AHB) examined the titles and abstracts of all identified studies. Full-text evaluations were performed on pertinent records to determine their eligibility. Any disagreements between the reviewers were settled through additional discussion and thorough examination.

### 2.2. Insulin Resistance Indices

TyG Index = ln (Fasting Triglycerides (mg/dL) × Fasting Glucose (mg/dL))/2

Please refer to reference [22].

HOMA-IR = Fasting Insulin (μU/mL) × Fasting Glucose (mg/dL)/405

Please refer to reference [21].

### 2.3. Data Extraction

Two independent authors (AS and AHB) conducted data extraction, resolving any disagreements through mutual agreement. The data gathered contained details regarding the first author’s name, the year of publication, the country of origin, the study design, the characteristics of the population, the sample size, and the main findings. The results were analyzed to identify key patterns, such as relationships between insulin resistance markers and FM symptoms. Differences in study designs, populations, and outcomes were examined to highlight inconsistencies. The review was purely qualitative, focusing on summarizing gaps and suggesting areas for further research.

## 3. Understanding Insulin Resistance

Insulin plays a critical role in the maintenance of energy balance and regulating glucose homeostasis throughout the body. Furthermore, it regulates the synthesis of the human sexual steroid hormone and influences its signal transduction at the cellular level. Insulin resistance is a metabolic disorder characterized by a reduced cellular response to insulin, which disrupts glucose uptake and leads to complex metabolic and hormonal imbalances. This condition results in elevated blood glucose levels and compensatory hyperinsulinemia as the pancreas seeks to mitigate the decreased insulin sensitivity. Chronic IR is often linked to obesity, type 2 diabetes, and several inflammatory disorders, such as hypertension, atherosclerosis, and steatotic liver disease [26,27]. Conversely, insulin also promotes anabolic processes and triggers transport systems, playing an important role in carbohydrate, protein, and lipid metabolism [28,29].

## 4. Insulin and Lipid Toxicity: Disruption of Cellular Functions

When cells are constantly subjected to high levels of insulin, there is a gradual reduction in insulin signaling. This condition, referred to as IR, occurs not only because of a decrease in the expression of insulin receptors on the surface of the cell but mainly because of dysfunction in insulin signal transduction. Extended exposure to high insulin levels leads to a reduction in autophosphorylation of the insulin receptor in comparison to brief insulin exposure, resulting in interruptions in the subsequent stages of the phosphoinositide 3-kinase (PI3K)–AKT signaling pathway [30,31,32]. As a result, in muscle and adipose cells, there is reduced AKT-stimulated translocation of glucose transporter type 4 (GLUT 4) to the cell surface. Therefore, IR can be viewed as a compensatory mechanism that limits excessive glucose uptake from the bloodstream, even in the context of chronically increased insulin levels. This helps to maintain glucose homeostasis in vivo while alleviating metabolic and oxidative stress associated with an excess of glucose [33,34,35,36]. Insulin toxicity is partially driven by the fact that elevated insulin levels trigger additional cellular responses that are not adequately suppressed during the state of IR. These responses contribute to the disruption of normal cellular function and exacerbate the metabolic disturbances associated with IR.

The severity and length of hyperglycemia contribute to the increased production of reactive nitrogen species (RNS) and reactive oxygen species (ROS), thus having an impact on several signaling pathways and aggravating oxidative disruption of lipids, nucleic acids, and proteins [37].

Prolonged elevated glucose levels in an IR state link with lipids and proteins and generate advanced lipoxidation end products and advanced glycation end products (AGEs), thus triggering oxidative stress [38].

Oxidative stress and lipid peroxidation play an important role in the pathogenesis of FM. ROS and RNS enhance the generation of products of lipid peroxidation, in parallel with increased cell mitophagy. Cell mitophagy causes a shift in the oxidant–antioxidant balanced milieu in favor of oxidation, thus leading to pain sensitivity and nerve and muscle dysfunction [39]. The induction of the inflammatory cascade by increased expression of pro-inflammatory cytokines further contributes to increased neuroinflammation and pain sensitivity [40]. Indeed, it was shown that metformin poses anti-inflammatory properties by activating AMPK and inhibiting the synthesis of TNF-α and IL-1β, thus diminishing neuroinflammation; this may enable therapeutic benefits mitigating FM pathology [40].

## 5. HOMA-IR: An Available Tool for Determining IR

HOMA-IR is a commonly utilized index for the estimation of insulin sensitivity using insulin and fasting glucose levels. HOMA-IR quantifies IR by multiplying these two concentrations and then dividing by a standard constant. Elevated HOMA-IR values suggest higher IR, i.e., diminished insulin sensitivity. This measure is commonly used in both clinical practice and research to evaluate the risk of metabolic diseases in several cardiovascular and non-cardiovascular diseases [41,42,43]. Its simplicity and ability to provide non-invasive insights into insulin sensitivity make HOMA-IR a valuable tool for monitoring metabolic health and guiding therapeutic strategies. However, there remains a lack of a universally accepted cutoff value for HOMA-IR in assessing IR. While several thresholds have been suggested for different populations, a value of 2.5 or higher is commonly used as a reference point to indicate significant IR [21,44]. This threshold helps differentiate between healthy and impaired glucose metabolism, though its applicability may vary across diverse groups.

HOMA-IR plays a pivotal role in evaluating IR across different medical conditions. For instance, a systematic review of 15 studies involving 655 treatment-naïve patients with acromegaly found that the average HOMA-IR was significantly elevated compared to the reference population, showing a mean difference of 2.04 (95% CI: 0.65–3.44, *p* < 0.01) [37]. Similarly, in obstructive sleep apnea (OSA), a meta-analysis of 16 case–control studies found a pooled standardized mean difference (SMD) of 0.51 (95% CI: 0.28–0.75, *p* < 0.001) in HOMA-IR between OSA patients and controls. Randomized controlled trials indicated that continuous positive airway pressure therapy reduced HOMA-IR by a statistically significant mean difference of −0.43 (95% CI: −0.75 to −0.11, *p* = 0.008), revealing its potential to reduce IR [45]. In the cancer field, a meta-analysis of 10 observational studies involving 1143 prostate cancer patients showed that fasting insulin levels were significantly higher than the controls, with a weighted mean difference (WMD) of 2.12 µIU/mL (95% CI: 0.26–3.99, *p* = 0.02). HOMA-IR differences between prostate cancer patients and controls were not statistically significant overall (WMD = 0.24, 95% CI: −0.56 to 1.04, *p* = 0.055) [46]. While the studies in the rheumatological area are limited, Müller et al. demonstrated that IR tends to emerge early in the rheumatoid arthritis (RA) disease course for most patients. It is observed more frequently in males and is linked to both the severity of RA and reduced appendicular lean muscle mass [47]. Nevertheless, research investigating the relationship between FM and HOMA-IR remains limited.

## 6. TyG Index: A More Available Method for Assessing IR

The TyG index is a method that integrates fasting TG and fasting plasma glucose levels into a straightforward, dimensionless index. This tool, which is commonly calculated through routine biochemical testing, has been validated for its positive association with IR and an increased risk of several diseases and conditions such as diabetes, as well as an elevated risk of metabolic and atherosclerotic cardiovascular diseases (ASCVDs) [48,49,50,51,52,53,54,55,56]. In a 2011 cross-sectional study conducted among 82 Brazilian individuals with type 2 diabetes mellitus (T2DM) or normal glucose tolerance, the TyG index was found to be a superior marker for estimation of IR compared to the established HOMA-IR index. Based on this study’s findings, the TyG index demonstrated a higher area under the receiver operating characteristic curve (AUC) of 0.79, as compared with 0.77 for the HOMA-IR index, indicating its greater accuracy in assessing insulin sensitivity [57]. Moreover, Lee et al. indicated that the TyG index demonstrated greater predictive power compared to the HOMA-IR index for the assessment of IR [58].

The association between the TyG index and health outcomes has been explored in many studies, emphasizing its role as a surrogate IR marker. Ding et al. [59] conducted a meta-analysis to find the correlation between the TyG index and ASCVDs, which include coronary artery disease (CAD) and stroke. The analysis, which included 8 cohort studies with a total of 5,731,294 participants, revealed that those with the highest TyG index faced a 61% greater risk of ASCVDs (HR 1.61, 95% CI 1.29–2.01, <0.001) and an elevated risk of CAD (HR 1.95, 95% CI 1.47–2.58) and stroke (HR 1.26, 95% CI 1.23–1.29) when compared to individuals with the lowest TyG index. Similarly, Beran et al. [60] explored the TyG index in relation to nonalcoholic fatty liver disease (NAFLD) in a meta-analysis of 17 studies involving 121,975 individuals. The results demonstrated that a higher TyG index was associated with a higher risk of NAFLD, with pooled odds ratios of 6.00 (95% CI 4.12–8.74) when the TyG index was assessed as a categorical variable and 2.25 (95% CI 1.66–3.04) when analyzed as a continuous variable.

Further research backs the connection between the TyG index and both cardiovascular and metabolic disorders. A systematic review was conducted to explore the association between the TyG index and peripheral artery disease (PAD). This investigation evaluated 22 studies involving 73,168 participants, demonstrating that individuals with PAD exhibited significantly elevated TyG levels than the control group (SMD 0.76, 95% CI 0.65–0.88, *p* < 0.001). Also, participants in the top quartile of TyG were 94% more likely to have PAD compared to those in the lowest quartile (OR 1.94, 95% CI 1.49–2.54, *p* < 0.001) [61]. Likewise, it was revealed that an increased TyG may be related to higher odds of hypertension in the adult population [62]. Moreover, it was determined that an elevated TyG index is associated with higher odds of cognitive decline [63]. These findings underscore the utility of the TyG index as a predictor of metabolic and cardiovascular diseases. Even so, the association of the TyG index and rheumatologic disorders has been less explored in the literature. In this regard, Yan et al. [64] revealed that the TyG index showed a positive association with arthritis among non-diabetic adults under 60 years old in the United States who were of normal weight. However, there is a lack of sufficient studies exploring the relationship between the TyG index and FM.

## 7. IR and Fibromyalgia

While the exact pathophysiology of FM is still not fully clear, the evidence points to a multifactorial origin. Metabolic disruptions, particularly IR and impaired glucose metabolism, are thought to play a significant role. This suggests that FM involves broader systemic dysfunction rather than being solely a pain-related condition, underscoring the importance of exploring these interactions to develop more effective treatment strategies [24,25]. Numerous studies have explored the connection between FM and IR. Research conducted in the United States indicated that women with FM were more prone to central obesity, elevated levels of glycated hemoglobin (HbA1c), higher serum TG, and increased blood pressure when compared to healthy individuals [65]. This study showed the first empirical confirmation that FM per se could be a risk factor for metabolic syndrome. However, the study included only 109 female participants and it cannot be generalized to both sexes.

Also, a national study conducted in Taiwan indicated that individuals with FM are at an increased risk for cardiometabolic comorbidities, such as hypertension, diabetes, dyslipidemia, and cardiovascular diseases [66]. The mentioned study [66] included a large sample of 61,612 patients with FM, thus enabling the subgroup analysis that confirmed the influence of FM on coronary heart disease (CHD). The long period of patient observation increased the ability of FM to predispose individuals to developing CHD. However, the limitation of the study was the inability to assess the correlation between the degree of pain and BMI, or assess the effect of smoking, given the fact that these are known risk factors for CHD or FM.

A study conducted in Spain found that patients with FM were more likely to have central obesity and reduced cardiopulmonary function than those without the condition [67]. In conclusion, an increasing amount of research suggests a possible correlation between IR and the onset and progression of FM. This highlights potential opportunities to better understand the underlying causes of FM and improve diagnostic methods.

In a study conducted by Fava et al. [68], the authors aimed to assess the metabolism of glucose and IR among 96 patients with FM, including those with and without cognitive impairment. The participants were split into two groups of those with and without memory deficits. They measured blood levels of glucose and insulin following a 2 h Oral Glucose Tolerance Test (OGTT), and IR was evaluated using the HOMA-IR formula. The results showed a significantly higher rate of glucose metabolism abnormalities in patients with memory deficits, with 79% of them exhibiting IR. Among these, 23% had impaired glucose tolerance, 4% were newly diagnosed with diabetes, and 52% had only IR. Obesity and overweight were more prevalent in those with memory deficits. The study found that IR, rather than body mass index (BMI) or waist-to-hip ratio, was associated with a higher risk of memory impairment. These findings suggest that IR could be a potential risk factor for memory deficits in individuals with FM. This study [68] was the first that examined the association between cognitive deficits and impaired glucose metabolism in patients with FM. However, it was conducted on the Italian population and could not be generalized to other ethnicities. Moreover, HOMA-IR was used as a surrogate marker of IR, instead of euglycemic–hyperinsulinemic clamp.

In an animal model study using Zucker Diabetic Fatty (ZDF) rats, a strong association was observed between chronic pain and IR. The model involved neuropathic thermal and mechanical hypersensitivity induced by chronic constriction injury of the sciatic nerve. The results showed that ZDF rats exhibited lower pain thresholds and slower recovery compared to Zucker Lean rats. Additionally, the progression of T2DM in ZDF rats was accelerated by nerve injury, leading to earlier hyperglycemia, more severe hyperinsulinemia, and higher HbA1c levels. Therefore, these suggest a bidirectional relationship between IR and chronic pain in this animal model, with impaired insulin receptor signaling possibly playing a role in this interaction [69].

García et al. demonstrated that fructose-induced IR can serve as a model for neuropathic pain in rats [70]. Their study revealed that IR triggered by fructose replicated key features of neuropathic pain, with nociceptive hypersensitivity attributed to the modulation of various ionic channels in primary afferent neurons. Advanced imaging techniques further showed that IR causes dysfunctions in brain microcirculation, leading to cerebral hypoperfusion [70]. Notably, similar focal deficits in brain perfusion have been observed in advanced imaging of patients with FM, highlighting a potential connection [71]. Another study was performed to investigate the prevalence of FM in patients with type 1 and T2DM and its potential relationship with DM disease control. They concluded that there was a higher prevalence of FM in patients with T2DM, but no link was found between FM prevalence and diabetes control [72]. Tishler et al. in a small sample size of 100 patients with diabetes also found that FM is frequently observed in patients with both type 1 and T2DM, with its occurrence potentially associated with the level of diabetes control [73].

All the mentioned points emphasize the connection between IR and chronic pain syndromes. Evidence suggests that IR can play a significant role in the onset and progression of chronic pain, particularly neuropathic pain. In line with animal models, clinical studies have also shown that metabolic disturbances, such as IR, can impact the nervous system, increasing pain sensitivity and exacerbating pain. These findings highlight the importance of IR in chronic pain syndromes.

### 7.1. Physiopathology of the Association Between IR and FM

The exact pathophysiology behind the association between IR and FM remains undetermined. However, one widely accepted hypothesis suggests that mitochondrial dysfunction and oxidative stress may be key factors contributing to this link [74]. A reduction in metabolic substrate oxidation seems to be a key defect that initiates a series of events, ultimately leading to intracellular diacylglycerol and ceramide accumulation, that disrupt insulin signaling and contribute to IR. Given the essential role of mitochondria in oxidative metabolism, they have been identified as the critical organelles connecting impaired fuel utilization, especially fatty acid oxidation, lipotoxicity, and IR. This connection between diminished mitochondrial oxidative capacity and IR has been found in significant studies showing mitochondrial dysfunction in individuals with T2DM. Early evidence linking mitochondrial dysfunction to IR comes from research on obese, insulin-resistant individuals who showed impaired lipid metabolism and reduced mitochondrial oxidative capacity in skeletal muscle compared to healthy, lean controls [75,76,77,78]. Additionally, numerous researchers have suggested that the loss of mitofusin2 (Mfn2), a protein located in the outer mitochondrial membrane that aids in mitochondrial fusion, leads to a decrease in mitochondrial coenzyme Q10 (CoQ10). This reduction in CoQ10 may disturb the mitochondrial respiratory chain by affecting mitochondrial uncoupling proteins and the mitochondrial permeability transition pore and heightening the production of reactive oxygen species. Moreover, CoQ10 has been associated with the regulation of serotonin levels and depressive symptoms in patients with FM. Although the precise role of Mfn2 in sustaining CoQ10 and mitochondrial function remains unclear, a study conducted by Mourier et al. [79] has revealed an unexpected function of Mfn2 in maintaining the terpenoid biosynthesis pathway, critical for CoQ10 production. Furthermore, the signaling pathway related to peroxisome proliferator-activated receptor gamma coactivator-1alpha (PGC-1α) has been demonstrated to affect Mfn2 expression, redirecting research focus from investigating the causes of FM to exploring potential strategies for symptom management [79,80,81,82].

### 7.2. Relationship Between TyG Index and HOMA-IR with FM

One of the studies that was performed to assess the role of the TyG index as an IR marker for FM employed a cross-sectional design to assess the correlation between IR and FM in a population of 140 female participants, divided into two groups: 70 diagnosed with FM and 70 age-matched healthy controls [83]. They showed that FM patients had a significantly higher TyG index (8.62 ± 0.59) compared to the control group (8.48 ± 0.44), with a *p*-value of 0.038, indicating a notable difference in metabolic risk between the two groups. Additionally, HOMA-IR values were significantly higher in FM patients (2.76 ± 2.59) than in the control group (1.72 ± 1.38), with a *p*-value of 0.002, further supporting the presence of greater IR in FM patients. The study also categorized FM patients based on their Fibromyalgia Impact Questionnaire—Revised (FIQR) scores, revealing that HOMA-IR values increased with the severity of FM—mild (1.50 ± 0.20), moderate (2.50 ± 0.50), and severe (3.50 ± 1.00)—all with *p*-values less than 0.05. These findings underscore the significant metabolic dysregulation present in FM patients compared to healthy controls [83]. Kim et al. designed a study aimed at assessing the relationship between FM, carotid arterial stiffness, and cardiometabolic risk factors [84]. In this study, 58 participants with FM and 158 healthy controls were analyzed. They indicated that FM patients exhibit significantly elevated TyG index values. The odds of high TG in FM patients were 4.73 times greater (95% CI: 2.29–9.79, *p* < 0.001), and impaired fasting glucose (IFG) was 4.27 times more likely (95% CI: 2.07–8.81, *p* < 0.001) compared to controls. These metabolic disruptions were correlated with arterial stiffness, as reflected by a significantly higher carotid beta-index in FM patients (mean beta-index: 10.6 [95% CI: 9.6–11.5]) versus controls (mean beta-index: 8.1 [95% CI: 6.8–9.5], *p* = 0.003). This indicates a loss of arterial elasticity in FM participants. However, the carotid intimal media thickness (cIMT) differences between FM and controls were not statistically significant (mean cIMT in FM: 0.70 mm vs. 0.65 mm, *p* = 0.145), suggesting that stiffness alterations occur without notable structural changes in the arterial wall. In addition, while correlations between the TyG index and the carotid beta index showed positive trends (r = 0.251, *p* = 0.072), statistical significance was not reached, indicating the need for larger-scale studies to confirm these findings [84].

The study conducted by Cure et al. investigates the interplay between FM and several metabolic markers, including IR, as measured by HOMA-IR and the TyG index [85]. The study analyzed the data from 176 FM patients and 184 controls, revealing key insights. While the TyG index did not differ significantly between FM patients (mean TyG = 3.72 ± 0.27) and controls (mean TyG = 3.75 ± 0.27; *p* = 0.369), FM patients demonstrated notable inflammatory and metabolic alterations. For instance, markers of systemic inflammation, such as erythrocyte sedimentation rate (ESR) and C-reactive protein (CRP), were significantly higher in FM patients (CRP: 2.9 vs. 2.1 mg/L, *p* = 0.001; ESR: 9 vs. 8 mm/h, *p* = 0.032) [79]. In another study [23] that included 33 FM patients, 13 had complete data to calculate the HOMA-IR and QUICKI (Quantitative Insulin Sensitivity Check Index) scores. All 13 patients (100%) exhibited abnormalities in at least one of the IR markers (HbA1c, HOMA-IR, or QUICKI), indicating the presence of IR within this subgroup. In addition, the authors concluded that the relationship between IR and FM was found to be unaffected by age, gender, or ethnicity [23].

Another study designed by Krupa et al. [86] found a correlation between IR, psychiatric issues, and treatment response to serotonin and noradrenaline reuptake inhibitors (SNRIs) in FM patients. A total of 59 patients with FM and 30 healthy controls were enrolled in the study. They concluded three main results: First, FM patients who did not respond to SNRI treatment (FM [T–]) had significantly higher levels of HOMA-IR, fasting insulin, and BMI compared to those who responded to SNRIs (FM [T+]) and healthy controls. Second, insulin resistance (HOMA-IR), depression, anxiety, and personality disorders were all predictors of poor response to SNRI treatment. Third, logistic regression models showed that HOMA-IR was one of the strongest predictors of treatment resistance, with an OR of 27.32 (95% CI, 4.72–157.7; *p* < 0.001), indicating a strong relationship between HOMA-IR and SNRI treatment resistance. Their results highlight the importance of targeting IR as a potential strategy for improving treatment outcomes in FM [86]. A summary of the articles referenced in Table 1 is provided.

### 7.3. Research Gaps and Future Research Directions

While mitochondrial dysfunction and oxidative stress are strongly involved in the connection between IR and FM, the exact mechanisms are still not fully understood. Further research is needed to clearly explain the role of mitochondrial proteins, such as Mfn2, and their impact on FM development and symptom management. Exploring the PGC-1α pathway and its influence on mitochondrial function could provide valuable insights into potential therapeutic strategies for managing FM-related symptoms.

## 8. Conclusions

This narrative review evaluated the complex relationship between IR and FM, a condition with an unclear etiology. However, emerging evidence indicates that metabolic disturbances, particularly IR, may have a pivotal role in FM development. The review highlights the utility of IR markers, the TyG index, and HOMA-IR in assessing metabolic health in FM patients. Several studies have shown that individuals with FM exhibit elevated levels of IR, which are associated with metabolic abnormalities like dyslipidemia, obesity, and impaired glucose tolerance. In contrast, no significant difference between patients with FM and healthy controls in terms of the TyG index was shown by some other studies. Despite this, IR has been implicated in the development of chronic pain conditions, such as FM, through mechanisms like mitochondrial dysfunction and oxidative stress. Although compelling evidence supports the link between IR and FM, significant gaps remain in understanding the precise mechanisms involved. More research is necessary to fully understand the underlying pathways of this association and determine whether targeting IR could provide new therapeutic options for FM. Large-scale, well-designed studies are needed to establish a definitive causal relationship and to identify potential biomarkers for early diagnosis of FM. In conclusion, this review emphasizes the critical need for further research into the metabolic factors, particularly IR, that influence FM, as addressing these factors could lead to improved management and better health outcomes for FM patients.

## Figures and Tables

**Table 1:** The Relationship Between Insulin Resistance markers and Fibromyalgia.

Author’s Name	Year	Design of Study	FM Population	N Total	Groups	Main Findings
Cure et al. [85]	2024	Retrospective, Cross-sectional	FM patients, Turkey	360	FM (n = 176), Controls (n = 184)	Elevated CRP, ESR, systemic inflammation; higher monocyte-HDL ratio and magnesium in controls; FM group had higher inflammatory markers, However, there was no significant difference between FM and controls regarding TyG, waist-height ratio, plasma atherogenic index, folate and vitamin B12 levels.
Tunç Karaman et al. [83]	2024	Cross-sectional	Female FM patients, Turkey	140	FM (n = 70), Controls (n = 70)	FM patients had higher HOMA-IR, fasting insulin, and TG; lower QUICKI; positive correlation of FM severity with metabolic indices.
Kim et al. [84]	2021	Retrospective, Cross-sectional	FM patients, Korea	216	FM (n = 58), Controls (n = 158)	FM associated with central obesity, higher TG, and impaired fasting glucose; advanced carotid arterial stiffness linked to IR.
Pappolla et al. [23]	2021	Cross-sectional observational study	FM patients, USA	33 FM patients	FM patients (33) vs. two control groups	13 FM patients with complete data had abnormal results in at least one IR marker (HbA1c, HOMA-IR, or QUICKI), indicating evidence of IR.
Krupa et al. [86]	2023	Cross-sectional observational study	FM Patients, Poland	89	FM Responders to SNRI treatment: 30 FM Non-responders to SNRI treatment: 29 Healthy Controls: 30	Insulin resistance (HOMA-IR) was a significant predictor of poor response to SNRI treatment.

## Supplementary Materials

The following supporting information can be downloaded at: https://www.mdpi.com/article/10.3390/diagnostics15040494/s1, Table S1: The Search strategy used for searching the databases.
