# Supplementary material for: Exploring the TyG Index and the Homeostasis Model Assessment of Insulin Resistance as Insulin Resistance Markers: Implications for Fibromyalgia Management and Understanding—A Narrative Review
Source: Diagnostics (Basel). 2025 Feb 18;15(4):494. doi: 10.3390/diagnostics15040494 (PMC11854647; doi:10.3390/diagnostics15040494)
Supplement: Supplementary file 1 [file diagnostics-15-00494-s001.zip › diagnostics-3455196-supplementary.pdf]

Supplementary Table S1. The Search strategy used for searching the databases

Database	Keywords/Terms	Number of Results
**PubMed**	("HOMA-IR*"[tiab] OR "Homeostatic Model Assessment*"[tiab] OR "Homair"[tiab] OR "homa ir"[tiab]) AND (("fibromyalgia"[Mesh] OR "fibromyalgia*"[tiab] OR "Fibromyalgias"[tiab] OR "Fibromyalgia-Fibromyositis Syndrome"[tiab] OR "Fibromyalgia Fibromyositis Syndrome"[tiab] OR "Fibromyalgia-Fibromyositis Syndromes"[tiab] OR "Syndrome, Fibromyalgia-Fibromyositis"[tiab] OR "Syndromes, Fibromyalgia-Fibromyositis"[tiab] OR "Rheumatism, Muscular"[tiab] OR "Muscular Rheumatism"[tiab] OR "Fibrositis"[tiab] OR "Fibrositides"[tiab] OR "Myofascial Pain Syndrome, Diffuse"[tiab] OR "Diffuse Myofascial Pain Syndrome"[tiab] OR "Fibromyositis-Fibromyalgia Syndrome"[tiab] OR "Fibromyositis Fibromyalgia Syndrome"[tiab] OR "Fibromyositis-Fibromyalgia Syndromes"[tiab] OR "Syndrome, Fibromyositis-Fibromyalgia"[tiab] OR "Syndromes, Fibromyositis-Fibromyalgia"[tiab] OR "Fibromyalgia, Secondary"[tiab] OR "Fibromyalgias, Secondary"[tiab] OR "Secondary Fibromyalgia"[tiab] OR "Secondary Fibromyalgias"[tiab] OR "Fibromyalgia, Primary"[tiab] OR "Fibromyalgias, Primary"[tiab] OR "Primary Fibromyalgia"[tiab] OR "Primary Fibromyalgias"[tiab]))	6
**Web of science**	TS=("HOMA-IR*" OR "Homeostatic Model Assessmen*" OR "Homair" OR "homa ir") AND TS=("fibromyalgia" OR "fibromyalgia*" OR "Fibromyalgias" OR "Fibromyalgia-Fibromyositis Syndrome" OR "Fibromyalgia Fibromyositis Syndrome" OR "Fibromyalgia-Fibromyositis Syndromes" OR "Syndrome, Fibromyalgia-Fibromyositis" OR "Syndromes, Fibromyalgia-Fibromyositis" OR "Rheumatism, Muscular" OR "Muscular Rheumatism" OR "Fibrositis" OR "Fibrositides" OR "Myofascial Pain Syndrome, Diffuse" OR "Diffuse Myofascial Pain Syndrome" OR "Fibromyositis-Fibromyalgia Syndrome" OR "Fibromyositis Fibromyalgia Syndrome" OR "Fibromyositis-Fibromyalgia Syndromes" OR "Syndrome, Fibromyositis-Fibromyalgia" OR "Syndromes, Fibromyositis-Fibromyalgia" OR "Fibromyalgia, Secondary" OR "Fibromyalgias, Secondary" OR "Secondary Fibromyalgia" OR "Secondary Fibromyalgias" OR "Fibromyalgia, Primary" OR "Fibromyalgias, Primary" OR "Primary Fibromyalgia" OR "Primary Fibromyalgias")	7
**Scopus**	TITLE-ABS-KEY ("HOMA-IR*" OR "Homeostatic Model Assessmen*" OR "Homair" OR "homa ir") AND TITLE-ABS-KEY ("fibromyalgia" OR "fibromyalgia*" OR "Fibromyalgias" OR "Fibromyalgia-Fibromyositis Syndrome" OR "Fibromyalgia Fibromyositis Syndrome" OR "Fibromyalgia-Fibromyositis Syndromes" OR "Syndrome, Fibromyalgia-Fibromyositis" OR "Syndromes, Fibromyalgia-Fibromyositis" OR "Rheumatism,	7

	Muscular" OR "Muscular Rheumatism" OR "Fibrositis" OR "Fibrositides" OR "Myofascial Pain Syndrome, Diffuse" OR "Diffuse Myofascial Pain Syndrome" OR "Fibromyositis-Fibromyalgia Syndrome" OR "Fibromyositis Fibromyalgia Syndrome" OR "Fibromyositis-Fibromyalgia Syndromes" OR "Syndrome, Fibromyositis-Fibromyalgia" OR "Syndromes, Fibromyositis-Fibromyalgia" OR "Fibromyalgia, Secondary" OR "Fibromyalgias, Secondary" OR "Secondary Fibromyalgia" OR "Secondary Fibromyalgias" OR "Fibromyalgia, Primary" OR "Fibromyalgias, Primary" OR "Primary Fibromyalgia" OR "Primary Fibromyalgias")	
**PubMed**	(("fibromyalgia"[Mesh] OR "fibromyalgia*"[tiab] OR "Fibromyalgias"[tiab] OR "Fibromyalgia-Fibromyositis Syndrome"[tiab] OR "Fibromyalgia Fibromyositis Syndrome"[tiab] OR "Fibromyalgia-Fibromyositis Syndromes"[tiab] OR "Syndrome, Fibromyalgia-Fibromyositis"[tiab] OR "Syndromes, Fibromyalgia-Fibromyositis"[tiab] OR "Rheumatism, Muscular"[tiab] OR "Muscular Rheumatism"[tiab] OR "Fibrositis"[tiab] OR "Fibrositides"[tiab] OR "Myofascial Pain Syndrome, Diffuse"[tiab] OR "Diffuse Myofascial Pain Syndrome"[tiab] OR "Fibromyositis-Fibromyalgia Syndrome"[tiab] OR "Fibromyositis Fibromyalgia Syndrome"[tiab] OR "Fibromyositis-Fibromyalgia Syndromes"[tiab] OR "Syndrome, Fibromyositis-Fibromyalgia"[tiab] OR "Syndromes, Fibromyositis-Fibromyalgia"[tiab] OR "Fibromyalgia, Secondary"[tiab] OR "Fibromyalgias, Secondary"[tiab] OR "Secondary Fibromyalgia"[tiab] OR "Secondary Fibromyalgias"[tiab] OR "Fibromyalgia, Primary"[tiab] OR "Fibromyalgias, Primary"[tiab] OR "Primary Fibromyalgia"[tiab] OR "Primary Fibromyalgias"[tiab])) AND (("TyG"[tw] OR "triglyceride glucose"[tw] OR "triglyceride-glucose"[tw] OR "triglyceride-glucose index"[tw] OR "triglyceride and glucose"[tw] OR "triglycerides-glucose"[tw] OR "triglycerides glucose"[tw]))	3
**Web of science**	(TS="fibromyalgia" OR TS="fibromyalgia*" OR TS="Fibromyalgias" OR TS="Fibromyalgia-Fibromyositis Syndrome" OR TS="Fibromyalgia Fibromyositis Syndrome" OR TS="Fibromyalgia-Fibromyositis Syndromes" OR TS="Syndrome, Fibromyalgia-Fibromyositis" OR TS="Syndromes, Fibromyalgia-Fibromyositis" OR TS="Rheumatism, Muscular" OR TS="Muscular Rheumatism" OR TS="Fibrositis" OR TS="Fibrositides" OR TS="Myofascial Pain Syndrome, Diffuse" OR TS="Diffuse Myofascial Pain Syndrome" OR TS="Fibromyositis-Fibromyalgia Syndrome" OR TS="Fibromyositis Fibromyalgia Syndrome" OR TS="Fibromyositis-Fibromyalgia Syndromes" OR TS="Syndrome, Fibromyositis-Fibromyalgia" OR TS="Syndromes, Fibromyositis-Fibromyalgia" OR TS="Fibromyalgia, Secondary" OR TS="Fibromyalgias, Secondary" OR TS="Secondary Fibromyalgia" OR TS="Secondary Fibromyalgias" OR TS="Fibromyalgia, Primary" OR TS="Fibromyalgias, Primary" OR TS="Primary Fibromyalgia" OR TS="Primary Fibromyalgias") AND (TS="TyG" OR TS="triglyceride glucose" OR TS="triglyceride-glucose" OR TS="triglyceride-glucose index" OR TS="triglyceride and glucose" OR TS="triglycerides-glucose" OR TS="triglycerides glucose")	2
**Scopus**	(TITLE-ABS-KEY ("fibromyalgia") OR TITLE-ABS-KEY ("fibromyalgia*") OR TITLE-ABS-KEY ("Fibromyalgias") OR TITLE-ABS-KEY ("Fibromyalgia-Fibromyositis Syndrome") OR TITLE-ABS-KEY ("Fibromyalgia Fibromyositis Syndrome") OR TITLE-ABS-KEY ("Fibromyalgia-Fibromyositis Syndromes") OR	2

	TITLE-ABS-KEY ("Syndrome, Fibromyalgia-Fibromyositis") OR TITLE-ABS-KEY ("Syndromes, Fibromyalgia-Fibromyositis") OR TITLE-ABS-KEY ("Rheumatism, Muscular") OR TITLE-ABS-KEY ("Muscular Rheumatism") OR TITLE-ABS-KEY ("Fibrositis") OR TITLE-ABS-KEY ("Fibrositides") OR TITLE-ABS-KEY ("Myofascial Pain Syndrome, Diffuse") OR TITLE-ABS-KEY ("Diffuse Myofascial Pain Syndrome") OR TITLE-ABS-KEY ("Fibromyositis-Fibromyalgia Syndrome") OR TITLE-ABS-KEY ("Fibromyositis Fibromyalgia Syndrome") OR TITLE-ABS-KEY ("Fibromyositis-Fibromyalgia Syndromes") OR TITLE-ABS-KEY ("Syndrome, Fibromyositis-Fibromyalgia") OR TITLE-ABS-KEY ("Syndromes, Fibromyositis-Fibromyalgia") OR TITLE-ABS-KEY ("Fibromyalgia, Secondary") OR TITLE-ABS-KEY ("Fibromyalgias, Secondary") OR TITLE-ABS-KEY ("Secondary Fibromyalgia") OR TITLE-ABS-KEY ("Secondary Fibromyalgias") OR TITLE-ABS-KEY ("Fibromyalgia, Primary") OR TITLE-ABS-KEY ("Fibromyalgias, Primary") OR TITLE-ABS-KEY ("Primary Fibromyalgia") OR TITLE-ABS-KEY ("Primary Fibromyalgias")) AND (TITLE-ABS-KEY ("TyG") OR TITLE-ABS-KEY ("triglyceride glucose") OR TITLE-ABS-KEY ("triglyceride-glucose") OR TITLE-ABS-KEY ("triglyceride-glucose index") OR TITLE-ABS-KEY ("triglyceride and glucose") OR TITLE-ABS-KEY ("triglycerides-glucose") OR TITLE-ABS-KEY ("triglycerides glucose"))	
